# Supplementary material for: Blue mussel shell shape plasticity and natural environments: a quantitative approach
Source: Sci Rep. 2018 Feb 12;8:2865. doi: 10.1038/s41598-018-20122-9 (PMC5809382; doi:10.1038/s41598-018-20122-9)
Supplement: Supplementary file 1 — Supplementary Information [file 41598_2018_20122_MOESM1_ESM.pdf]

Blue mussel shell shape plasticity and natural environments: a quantitative approach

Luca Telesca[1,2,*], Kati Michalek[3,+], Trystan Sanders[4,+], Lloyd S. Peck[2], Jakob Thyrring[5,6], and Elizabeth M. Harper[1,*]

[1]Department of Earth Sciences, University of Cambridge, CB2 3EQ Cambridge, United Kingdom
[2]British Antarctic Survey, CB3 0ET Cambridge, United Kingdom
[3]Scottish Association for Marine Science, PA37 1QA Oban, United Kingdom
[4]GEOMAR Helmholtz Centre for Ocean Research, 24148 Kiel, Germany
[5]Department of Bioscience, Arctic Research Centre, Aarhus University, 8000 Aarhus C, Denmark
[6]Department of Bioscience, Marine Ecology, Aarhus University, 8600 Silkeborg, Denmark
[+]these authors contributed equally to this work
[*]corresponding authors L.T (lt401@cam.ac.uk) and E.M.H. (emh21@cam.ac.uk)

SUPPLEMENTARY INFORMATION

List of Supplementary Figures

Figure S1 . . . 2
Figure S2 . . . 3
Figure S3 . . . 4
Figure S4 . . . 5
Figure S5 . . . 6
Figure S6 . . . 7
Figure S7 . . . 8
Figure S8 . . . 9
Figure S9 . . . 10

List of Supplementary Tables

Table S1 . . . 11
Table S2 . . . 12
Table S3 . . . 12
Table S4 . . . 13

Supplementary Contents

Supplementary Document S1 . . . 14
Supplementary Methods . . . 18
Supplementary References **20**

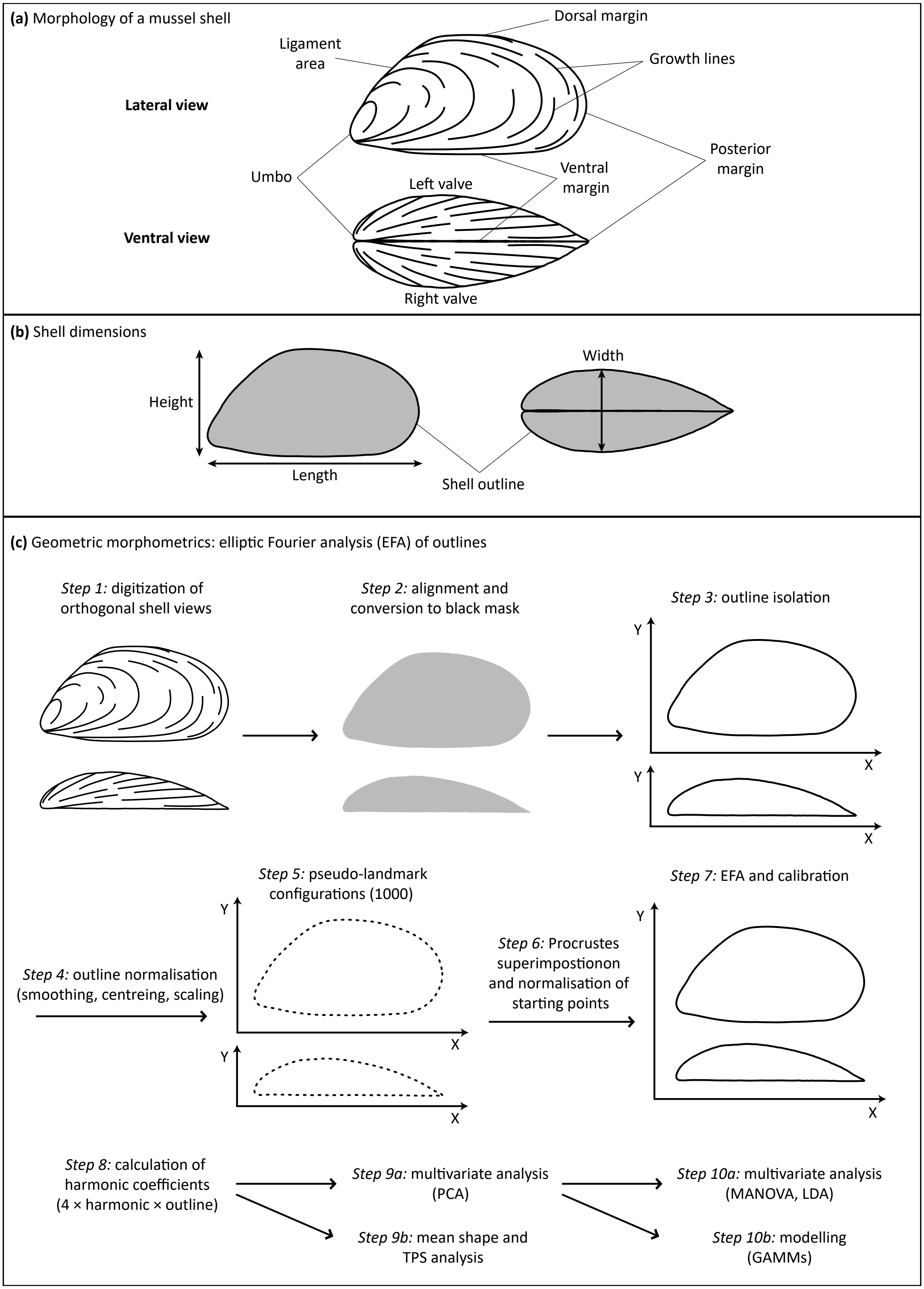

Figure S1. Blue mussel shell morphology and shape analysis. (**a**) Terminology used to describe the morphology of *Mytilus* shells. (**b**) Main shell linear dimensions. (**c**) Summary of the EFA of blue mussel shell outlines with details and graphical steps on the shape acquisition, processing and analysis of both lateral and ventral views.

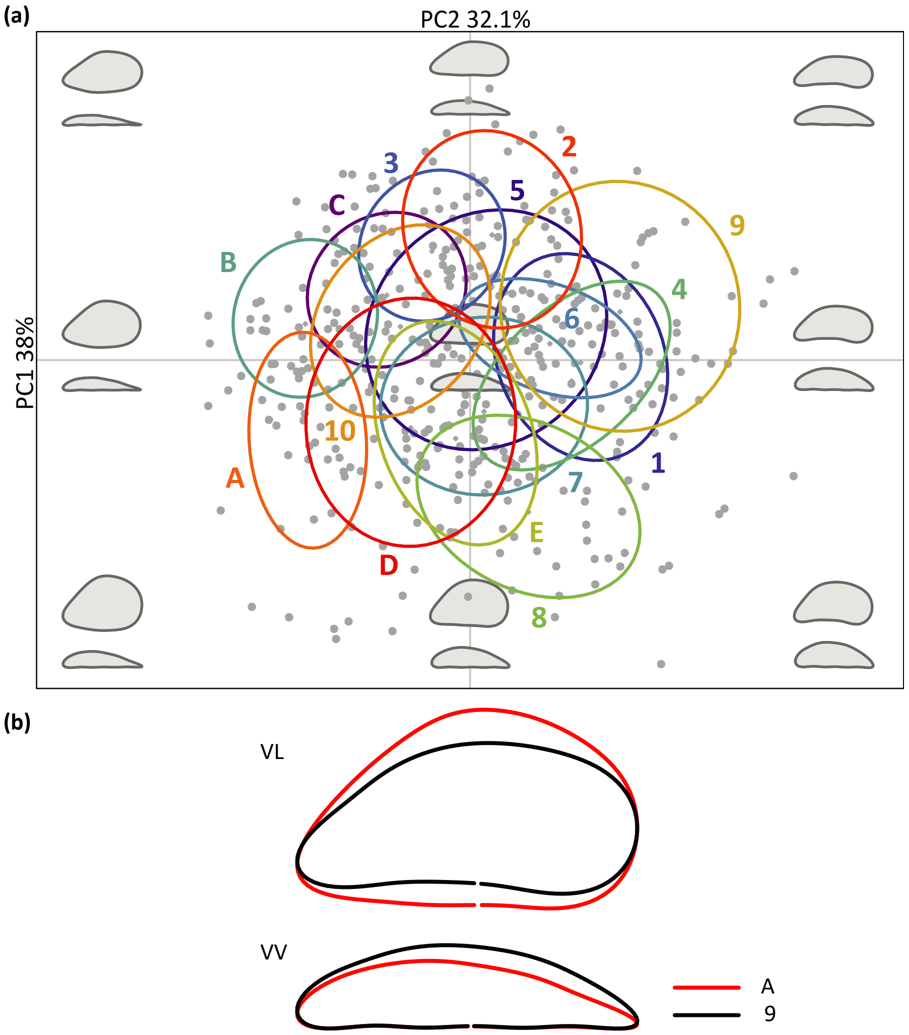

Figure S2. **Variation in outlines and shape features (Atlantic system).** (**a**) Scatterplots of the first two PCs from a PCA performed on the elliptic Fourier coefficients, of lateral and ventral shell views, showing a clear separation and marked shape variation among specimens from the pooled locations. Confidence intervals for each group and the reconstructed morphospace (background) were represented. (**b**) Mean shape differences of lateral (VL) and ventral (VV) views between populations at the extremes of the morphospace. Population A showed rounder and narrower shells, with bigger height and more convex ventral sides than population 9 with elongated, curved and wide shells.

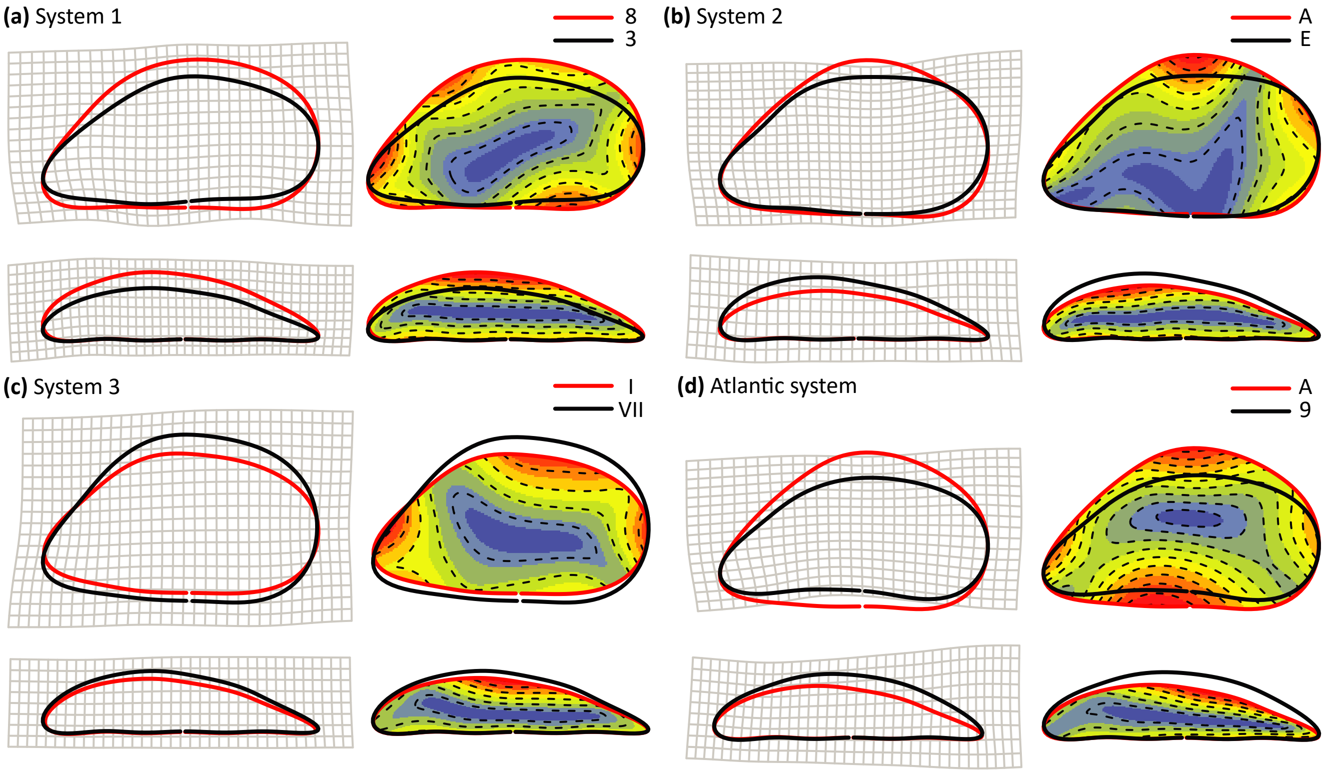

Figure S3. Mean shape differences at the extremes of the morphospace. Deformation grids (left), depicting the bindings required to pass from an extreme of the morphospace to another, and iso-deformation lines (right), representing the outline regions subjected to different degrees of change (blue: low deformation; red: strong deformation), for (**a**) System 1, populations 8 and 3, (**b**) System 2, populations A and E, (**c**) System 3, batches I and VII, and (**d**) Atlantic system, populations A and 9.

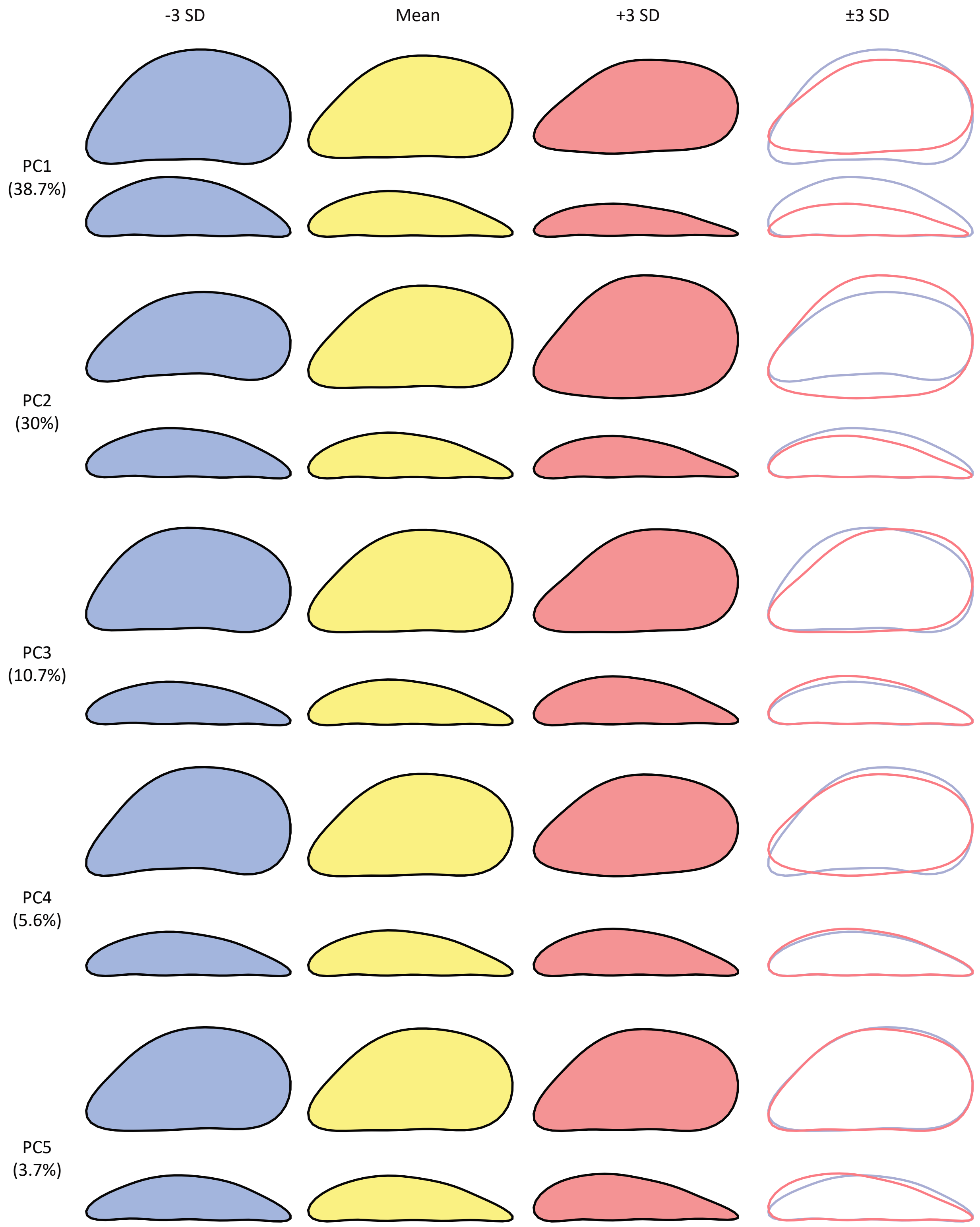

Figure S4. PCs contribution to shape reconstruction (System 1). Contribution of the first five shape variables (PCs) to shape variation (large-scale study system). The average shell shapes, for both lateral and ventral views, were represented for increasing values along each PC (−3 SD, Mean, +3 SD) and extreme shapes were compared (±3 SD).

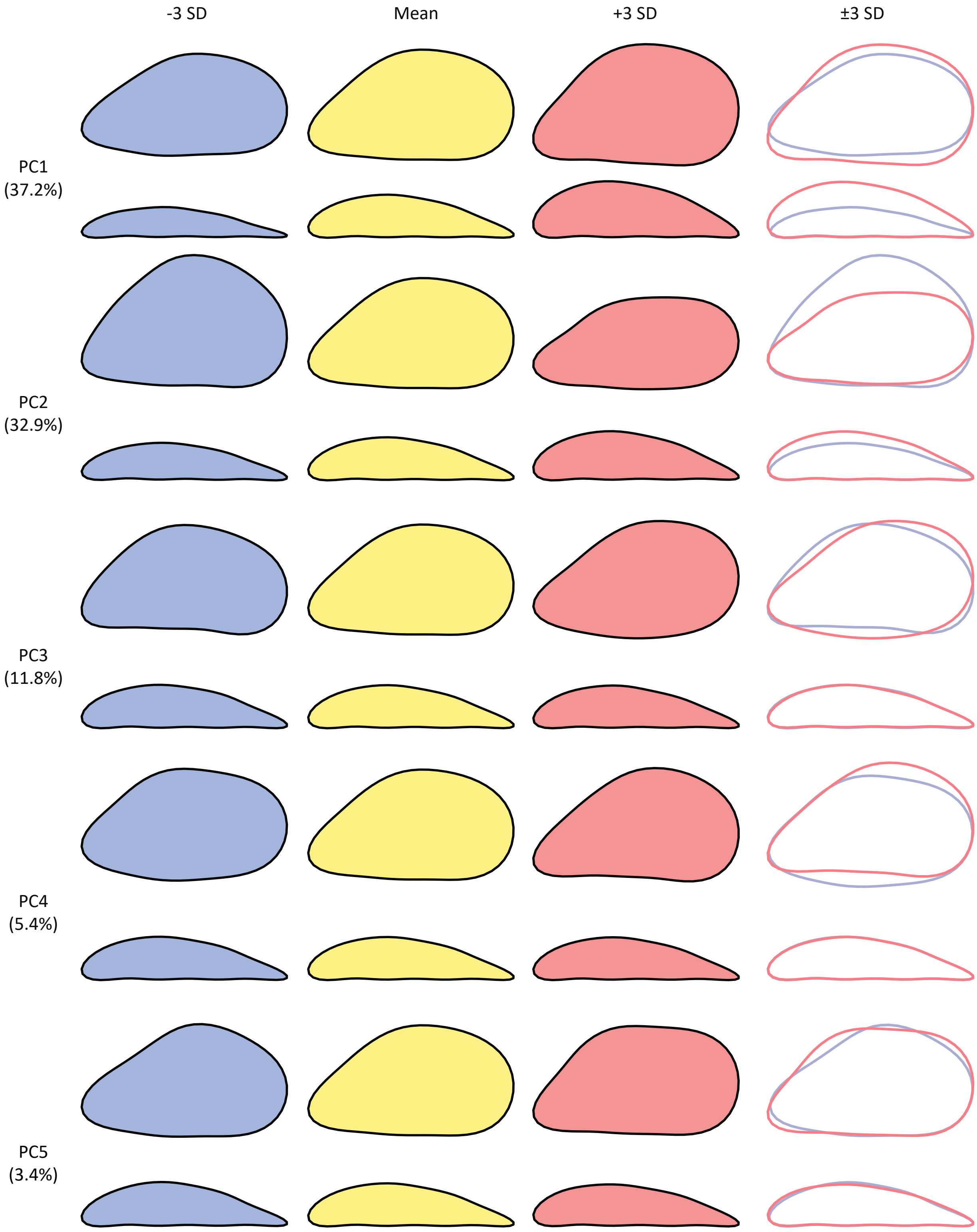

Figure S5. PCs contribution to shape reconstruction (System 2). Contribution of the first five shape variables (PCs) to shape variation (medium-scale study system). The average shell shapes, for both lateral and ventral views, were represented for increasing values along each PC (−3 SD, Mean, +3 SD) and extreme shapes were compared (±3 SD).

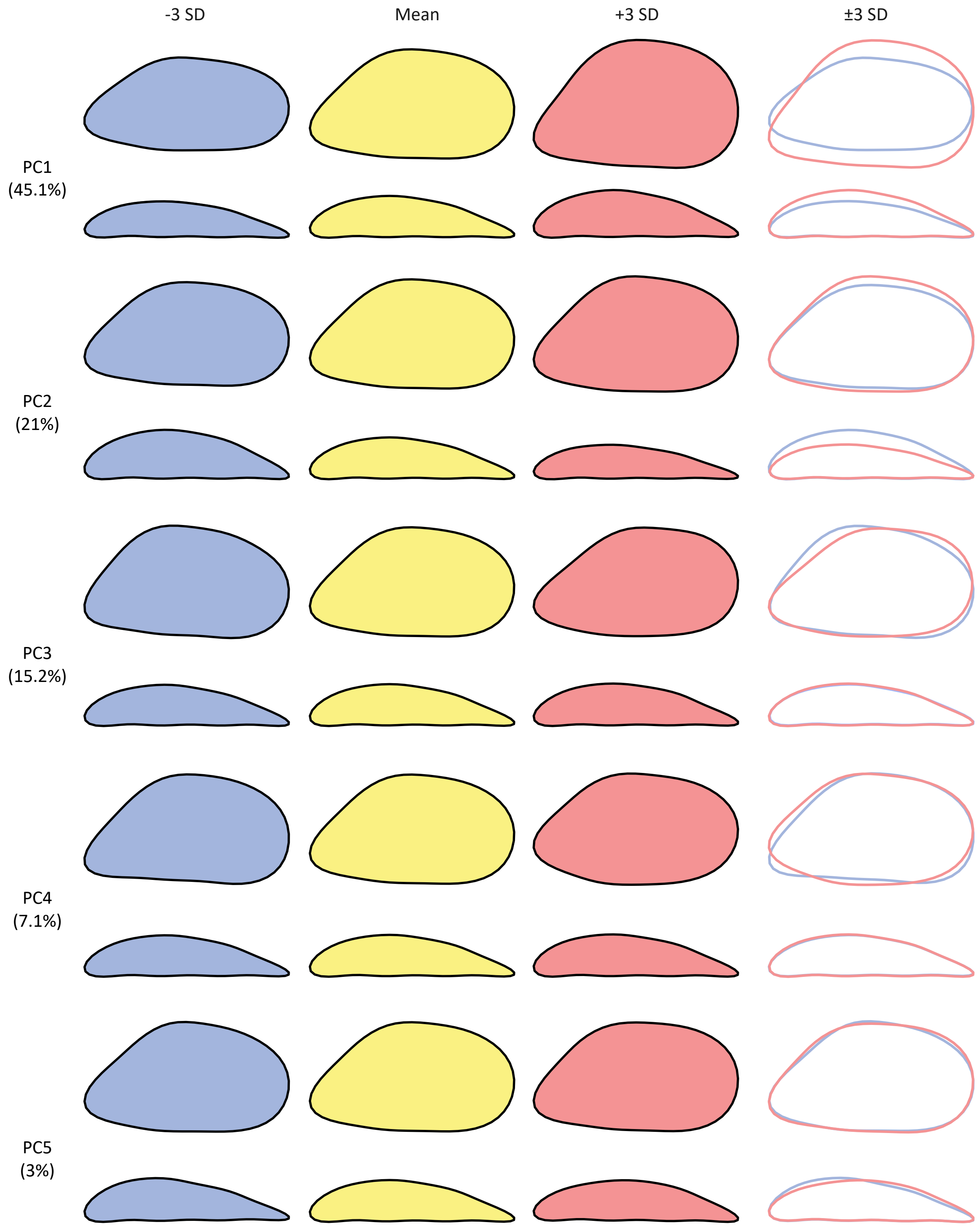

Figure S6. PCs contribution to shape reconstruction (System 3). Contribution of the first five shape variables (PCs) to shape variation (small-scale study system). The average shell shapes, for both lateral and ventral views, were represented for increasing values along each PC (−3 SD, Mean, +3 SD) and extreme shapes were compared (±3 SD).

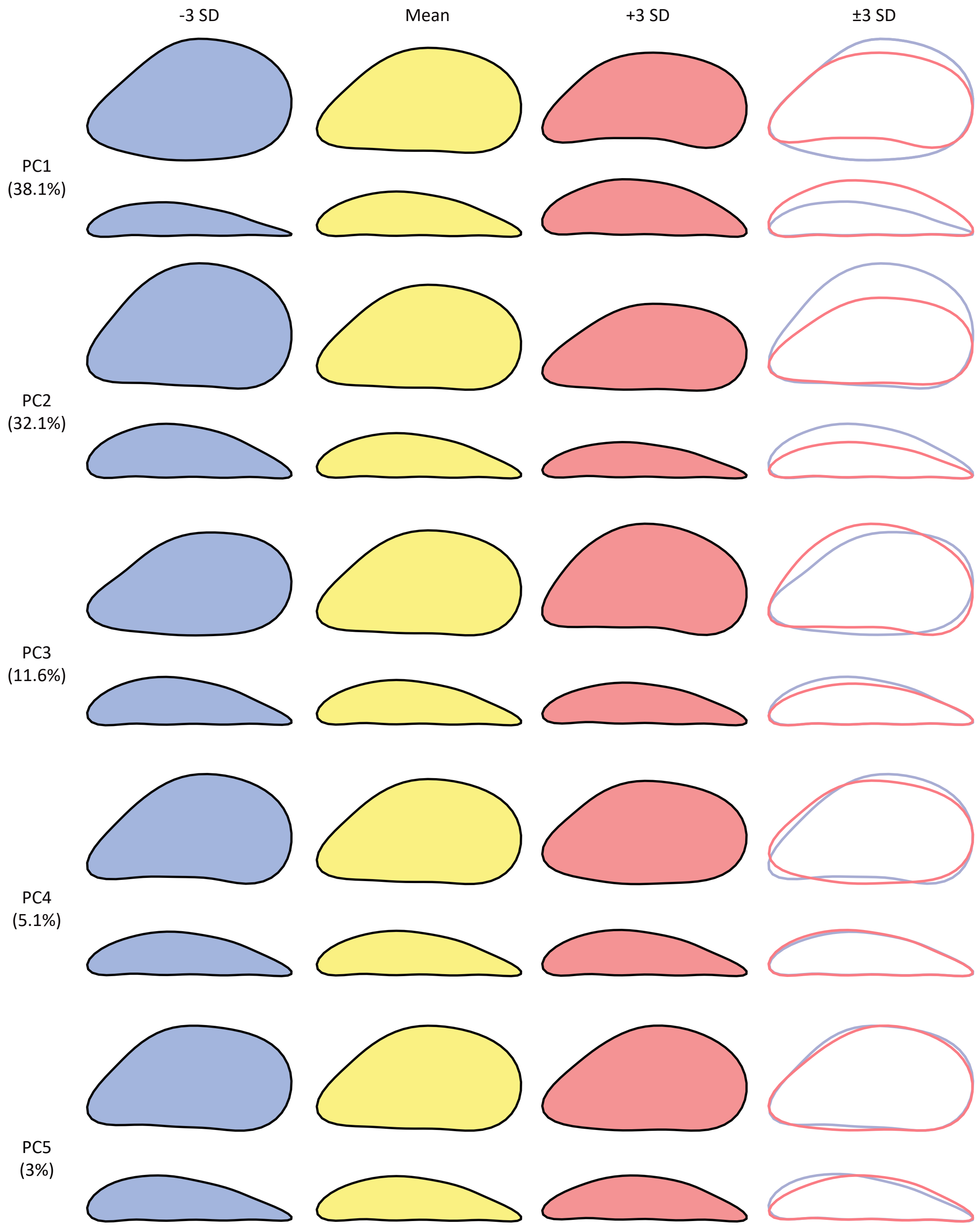

Figure S7. PCs contribution to shape reconstruction (Atlantic system). Contribution of the first five shape variables (PCs) to shape variation (pooled locations). The average shell shapes, for both lateral and ventral views, were represented for increasing values along each PC (−3 SD, Mean, +3 SD) and extreme shapes were compared (±3 SD).

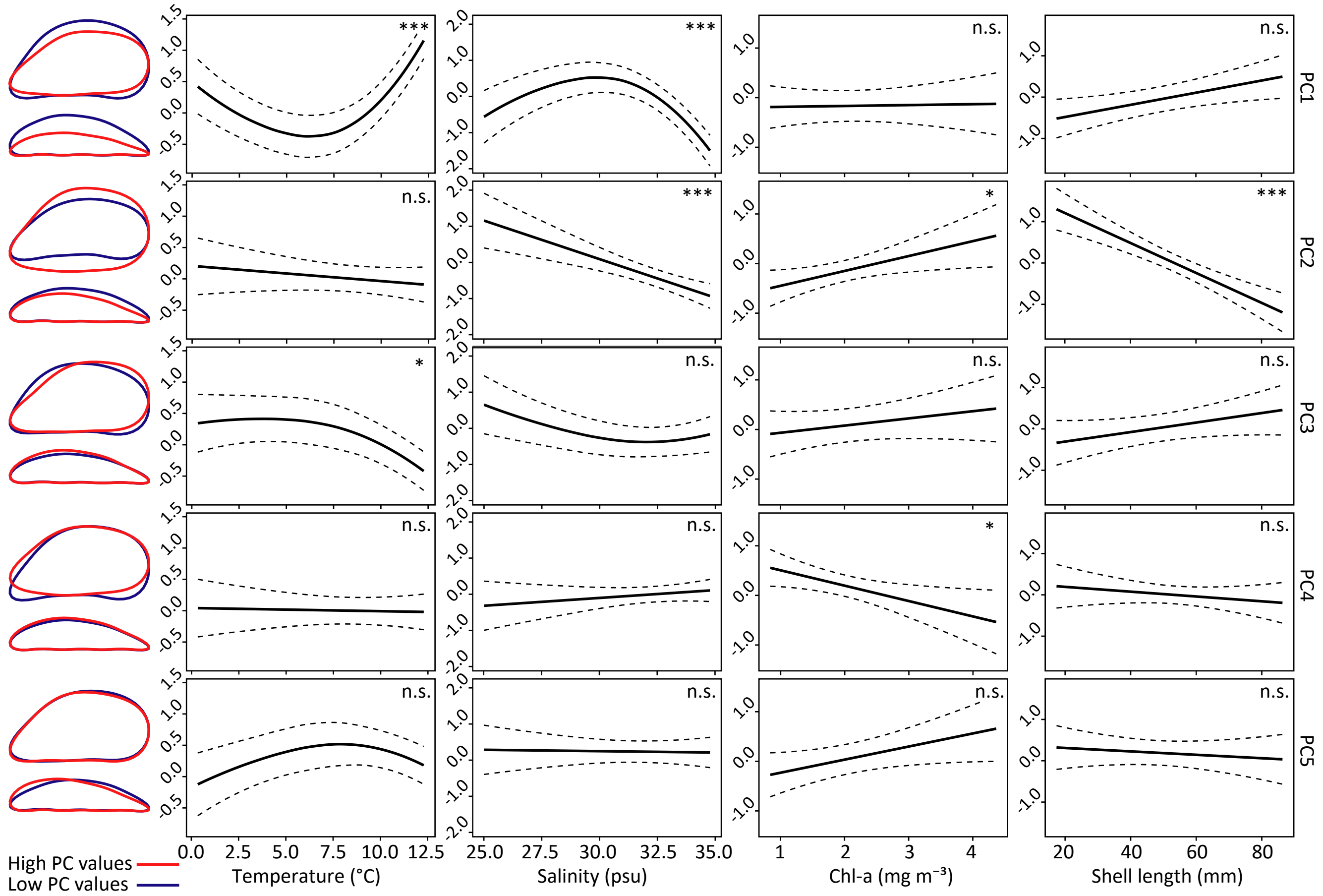

Figure S8. ***Mytilus* shell shape patterns (System 1).** Modelled shape trends of individual shell features (PC1-5) with environmental descriptors (surface waters temperature, salinity and chl-a concentration) and shell length (size) for the large-scale study system. Predicted values (continuous lines), 95%CI (dashed lines) and significance level of each fitted smoother and linear predictor are shown. Mussel shape variations described by each shape variable are represented through comparison of mean outlines reconstructed for low and high PC values (blue: Mean − 3 SD; red: Mean + 3 SD). (Significance, n.s. $p > 0.01$, * $p < 0.01$, ** $p < 0.001$, *** $p < 0.0001$)

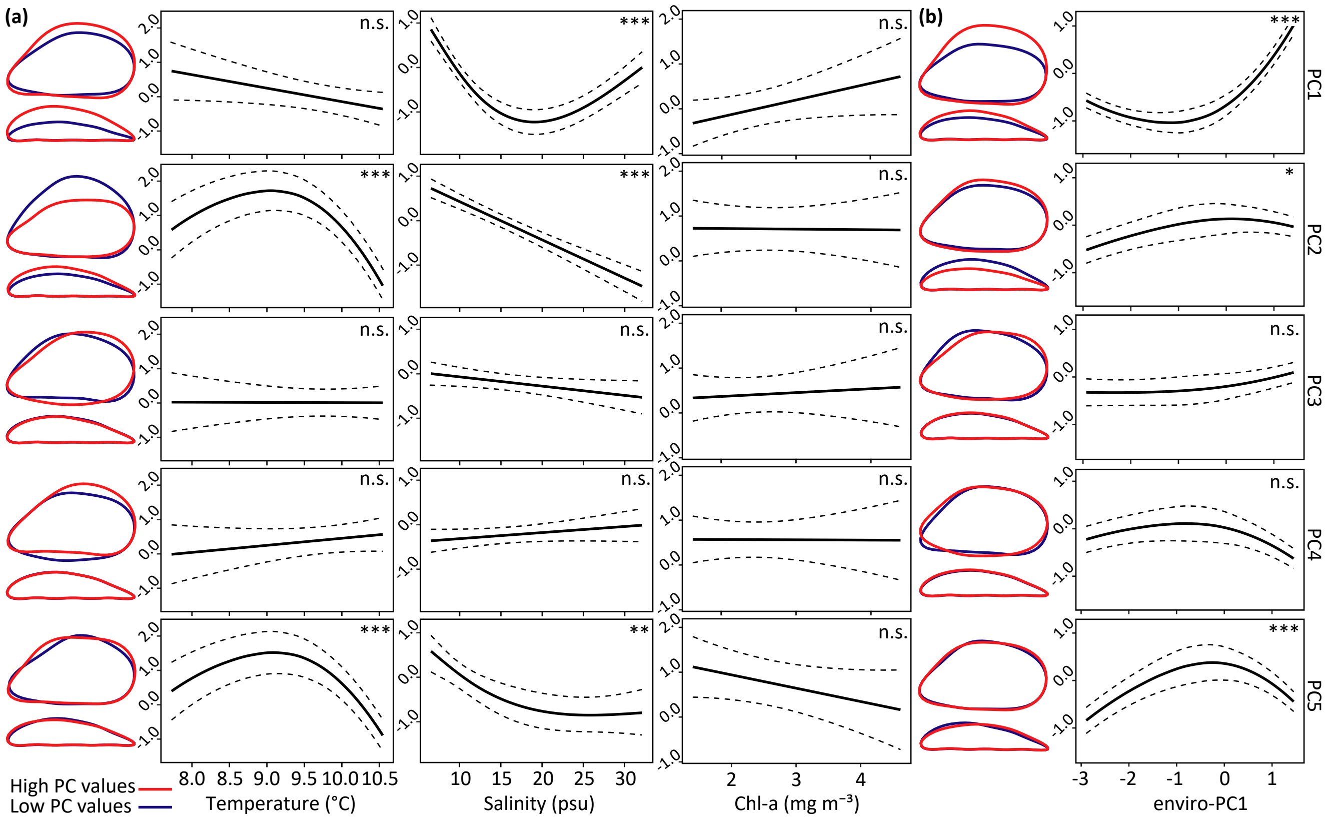

Figure S9. ***Mytilus*** **shell shape patterns (System 2 and 3).** Modelled shape trends of individual shell features (PC1-5) with environmental descriptors (surface waters temperature, salinity and chl-a concentration) and shell length (size) for the (**a**) medium- and (**b**) small-scale study systems. Predicted values (continuous lines), 95%CI (dashed lines) and significance level of each fitted smoother and linear predictor are shown. Mussel shape variations described by each shape variable are represented through comparison of mean outlines reconstructed for low and high PC values (blue: Mean − 3 SD; red: Mean + 3 SD). (Significance, n.s. $p > 0.01$, * $p < 0.01$, ** $p < 0.001$, *** $p < 0.0001$)

System	Location	ID	*n*	Longitude	Latitude	Status of mussels	Reference to taxonomic status of populations and/or previously published use of the shells
System 1	Exmouth (England, UK)	1	30	3°25′44.8″W	50°37′19.2″N	Me	Hilbishet *et al.* (2002)[1]<br>Annual monitoring 2015
	Oostende (Belgium)	2	30	2°54′15.6″E	51°13′49.4″N	Me	Kijewski *et al.* (2011)[2]<br>Kijewski *et al.* (2009)[3]
	Texel (Netherlands)	3	30	4°47′46.2″E	53°0′19.3″N	94%Me, 6%Me×Mg	Kijewski *et al.* (2011)[2]<br>Kijewski *et al.* (2009)[3]
	Menai Bridge (Wales, UK)	4	30	4°15′20.8″W	53°10′22.7″N	Me	Kijewski *et al.* (2009)[3]<br>Annual monitoring 2015
	Tarbet (Scotland, UK)	5	30	5°24′40.8″W	55°51′56.0″N	Me	Yarra *et al.* (2016)[4]
	St. Andrews (Scotland, UK)	6	30	2°46′53.3″W	56°20′22.6″N	Me	Tyler-Walters *et al.* (2016)[5]<br>MPA monitoring (JCNN, SNH)
	Kristineberg (Sweden)	7	30	11°25′19.5″E	58°12′37.8″N	94%Me, 6%Me×Mt	Stuckas *et al.* (2017)[6]<br>Brooks & Farmed (2013)[7]<br>Breusing (2012)[8]<br>Väinölä & Strelkov (2011)[9]<br>Kijewski *et al.* (2011)[2]<br>Stuckas *et al.* (2009)[10]
	Tromsø (Norway)	8	31	19°8′36.8″E	70°4′28.8″N	Me	Mathiesen *et al.* (2017)[11]<br>Brooks & Farmed (2013)[7]<br>Väinölä & Strelkov (2011)[9]
	Upernavik (Greenland)	9	28	56°6′10.1″W	72°47′38.0″N	33% Me, 51%Mt, 14%Me×Mt	Mathiesen *et al.* (2017)[11]
	Qaanaaq (Greenland)	10	28	69°14′25.1″W	77°27′54.0″N	Mt	Mathiesen *et al.* (2017)[11]
System 2	Sylt (Germany)	A	28	8°26′9.4″E	55°1′32.0″N	Me	Stuckas *et al.* (2017)[6]<br>Breusing (2012)[8]<br>Väinölä & Strelkov (2011)[9]<br>Stuckas *et al.* (2009)[10]<br>Bierne *et al.* (2003)[12]
	Kiel (Germany)	B	25	10°8′56.0″E	54°19′45.1″N	68%Me, 32%Me×Mt	Stuckas *et al.* (2017)[6]<br>Breusing (2012)[8]<br>Stuckas *et al.* (2009)[10]
	Ahrenshoop (Germany)	C	30	12°25′37.0″E	54°23′12.7″N	80%Me, 20%Me×Mt	Stuckas *et al.* (2017)[6]<br>Kijewski *et al.* (2011)[2]<br>Stuckas*et al.* (2009)[10]
	Usedom (Germany)	D	28	14°0′39.7″E	54°3′20.2″N	37%Me, 10%Mt, 53%Me×Mt	Stuckas *et al.* (2017)[6]<br>Kijewski*et al.* (2006)[13]
	Nynäshamn (Sweden)	E	27	17°55′45.4″E	58°52′38.5″N	Mt	Stuckas *et al.* (2017)[6]<br>Breusing (2012)[8]<br>Väinölä & Strelkov (2011)[9]<br>Kijewski *et al.* (2011)[2]<br>Stuckas *et al.* (2009)[10]
System 3	Loch Leven (Scotland, UK)	X	120	5°1′41.2″W	56°42′35.1″N	90%Me, 1%Mt, 3%Me×Mt, 6%Me×Mg	Dias *et al.* (2009)[14]

Table S1. Origin and details of mussel specimens. Information on collection areas and taxonomic status of the *Mytilus* populations used for the elliptic Fourier analysis of outlines. For each group, the study system, geographical location, population identifier (ID), samples size (*n*), site coordinates (longitude and latitude), genotypic status (Me: *Mytilus edulis*; Mt: *M.trossulus*; Mg: *M.galloprovincialis* and hybrids), and reference and/or previous use of the studied populations are reported.

System	Selected final model			
Atlantic	$SV_{ijk} =$	$\alpha_j + f_j(Temperature_i) + f_j(Salinity_i) + f_j(Chla_i) + \beta_{1j}Length_{ik} + Site_i + \varepsilon_{ijk}$	$Site_i \sim N(0;\sigma^2_{site})$	$\varepsilon_{ijk} \sim N(0;\sigma^2 \times e^{2\delta_j Temperature_i})$
System 1	$SV_{ijk} =$	$\alpha_j + f_j(Temperature_i) + f_j(Salinity_i) + \beta_{1j}Chla_i + \beta_{2j}Length_{ik} + Site_i + \varepsilon_{ijk}$	$Site_i \sim N(0;\sigma^2_{Site})$	$\varepsilon_{ijk} \sim N(0;\sigma^2 \times e^{2\delta_j Temperature_i})$
System 2	$SV_{ijk} =$	$\alpha_j + f_j(Salinity_i) + \beta_1 Chla_i + \beta_2 Length_{ik} + Site_i + \varepsilon_{ijk}$	$Site_i \sim N(0;\sigma^2_{Site})$	$\varepsilon_{ijk} \sim N(0;\sigma^2 \times e^{2\delta_j Chla_i})$
	$SV_{ijk} =$	$\alpha_j + f_j(Temperature_i) + \beta_{1j}Chla_i + \beta_2 Length_{ik} + Site_i + \varepsilon_{ijk}$	$Site_i \sim N(0;\sigma^2_{Site})$	$\varepsilon_{ijk} \sim N(0;\sigma^2 \times e^{2\delta_j Chla_i})$
System 3	$SV_{ijk} =$	$\alpha_j + f_j(enviro.PC1_i) + Batch_i + \varepsilon_{ijk}$	$Batch_i \sim N(0;\sigma^2_{Batch})$	$\varepsilon_{ijk} \sim N(0;\sigma^2 \times e^{2\delta_j enviro.PC1_i})$

Table S2. Optimal models. Best models after selection of random part (variance structures) and fixed components for each study systems are reported.

System	Factor	*df*	Wilks' λ	approx. *F*	num. *df*	den. *df*	*p*-value
Atlantic	Site	14	0.032	12.61	140	3381	<0.0001
	Length	1	0.873	5.95	10	410	<0.0001
System 1	Site	9	0.059	10.88	90	1889	<0.0001
	Length	1	0.849	4.94	10	277	<0.0001
System 2	Site	4	0.049	14.20	40	468	<0.0001
	Length	1	0.872	1.80	10	123	0.068
System 3	Batch	3	0.186	2.17	30	311	<0.0001
	Length	1	0.830	8.04	10	106	0.025

Table S3. MANOVA output. Summary of MANOVA models on the first 10 calculated shape variables (PCs) for each study system, showing the effects of location of origin and shell length (size) on shape variance.

System	PC	% Variance	Contribution to the shell shape
System 1	PC1	38.7	Variation in shell height, ligament angle, ventral margin shape and shell width: high values corresponded to elongated and narrow shells, with acute ligament angles and convex ventral margins; low values to round and wide shells, with big ligament angles and concave ventral margins.
	PC2	30.0	Variation in the shape of ventral margin, ligament angle and shell height, with small contribution to the symmetry of ventral profile: low values corresponded to curved shells, with concave ventral margins, small ligament angles and symmetric ventral profiles; high values corresponded to round shells, with big ligament angles, convex ventral margins and less symmetric ventral profiles.
	PC3	10.7	Variation in the shape of umbo, ligament and ventral margin: low values corresponded to ”curved” shells with concave ventral margins and ligaments, and an umbo oriented towards the ventral side; high values described elliptical shells with convex ventral margins and ligaments, and an umbo oriented toward the anterior side.
	PC4	5.6	Variation in the shape of ventral margin and differences between elliptical and “curved” shells, with a concave ventral margin.
	PC5	3.7	Small variations in dorsoventral shape (more or less parallel margins) and the symmetry of ventral view outlines.
System 2	PC1	37.2	Variation in shell height, convexity of the ventral margin and shell width, and differences between elongated and narrow shells, and round and wide specimens with flat ventral margins.
	PC2	32.9	Variation in dorsal margin, ligament area and shell width: low values corresponded to large and narrow shells, with a round dorsoposterior commissure; high values indicated elongated and wide shells, with more parallel dorsoventral margins.
	PC3	11.8	Variation in the shape of ventral and dorsal margins, and differences between ”curved” and elliptical shells.
	PC4	5.4	Variation in ligament length, ventral and dorsal margin shape, showing a progressive increase of ventral margin convexity and ligament length for higher values.
	PC5	3.4	Variations in the shape of the dorsoventral margins (more or less parallel) and ligament length.
System 3	PC1	45.1	Variation in shell height, width and ligament: low values corresponded to elongated and narrow shells, and acute ligament angles; high values were associated with round and wide shells, with a big height and ligament angle.
	PC2	21.0	Variation in shell height and width: increasing values corresponded to increasing shell height and decreasing width.
	PC3	15.2	Variation in ligament area, dorsoposterior and ventral margins shape: low values corresponded to a big ligament angle; high values indicated increasing roundness of posterior margins and a more acute ligament angle.
	PC4	7.1	Variation in the shape of ventral margin and differences between ”curved” shells with concave ventral margins and elliptical shells.
	PC5	3.0	Variation in dorsoposterior margins shape and the symmetry of ventral view.

Table S4. PCs contribution to the shell shape. Proportion of shape variance captured by individual shape variables and description of their contributions to the shell features and mean shape reconstruction for each study system (Supplementary Figure S5, S6, S7).

Supplementary Document S1

Information on the environmental parameters investigated (water temperature, salinity and chl-a concentration) for System 1, 2, and 3. Details on datasets used for measurement/calculation of mean annual values are provided.

System 1 and System 2 – Modelled datasets

List of the datasets used for the calculation of mean annual values of environmental descriptors. This study has been conducted using the Copernicus Marine Service Products: COPERNICUS – Marine Environment Monitoring System (http://marine.copernicus.eu/).

DATASET #1

Product name	**BALTIC SEA PHYSICS ANALYSIS AND FORECAST**
Product identifier	BALTICSEA_ANALYSIS_FORECAST_PHYS_003_006
Link	http://marine.copernicus.eu/services-portfolio/access-to-products/?option=com_csw&view=details&product_id=BALTICSEA_ANALYSIS_FORECAST_PHY_003_006 (last accessed on 15-12-2017)
Short description	This Baltic Sea physical model product provides forecasts for the physical conditions in the Baltic Sea. The Baltic forecast is updated twice daily providing a new two days forecast with hourly data for sea level variations, ice concentration and thickness at the surface, and temperature, salinity and horizontal velocities for the 3D field. The product is based on the 3D ocean model code HBM developed within the Baltic ocean community.
Spatial resolution	0.08 degree
Vertical coverage	from -5500.0 m to 0.0 m
Temporal resolution	Daily mean, hourly mean
Update frequency	Daily
Production unit	GLO-MERCATOR-TOULOUSE-FR

DATASET #2

Product name	**BALTIC SEA BIOGEOCHEMISTRY ANALYSIS AND FORECAST**
Product identifier	BALTICSEA_ANALYSIS_FORECAST_BIO_003_007
Link	http://marine.copernicus.eu/services-portfolio/access-to-products/?option=com_csw&view=details&product_id=BALTICSEA_ANALYSIS_FORECAST_BIO_003_007 (last accessed on 15-12-2017)
Short description	This Baltic Sea biogeochemical model product provides forecasts for the biogeochemical conditions in the Baltic Sea. The Baltic forecast is updated twice daily providing a new two days forecast with hourly data for the parameters dissolved oxygen, nitrate, phosphate, chl-a. The product is produced by the biogeochemical model ERGOM one way coupled to the Baltic 3D ocean model HBM, which provides the CMEMS Baltic physical ocean forecast product.
Spatial resolution	2 km
Vertical coverage	from -400 m to 0 m
Temporal resolution	Daily mean, hourly instantaneous
Update frequency	Daily
Production unit	BAL-DMI-COPENHAGEN-DK

DATASET #3

Product name	**GLOBAL OCEAN 1/12°PHYSICS ANALYSIS AND FORECAST UPDATED DAILY**
Product identifier	GLOBAL_ANALYSIS_FORECAST_PHY_001_024
Link	`http://marine.copernicus.eu/services-portfolio/access-to-products/?option=com_csw&view=details&product_id=GLOBAL_ANALYSIS_FORECAST_PHY_001_024` (last accessed on 15-12-2017)
Short description	The Operational Mercator global Ocean analysis and forecast system at 1/12 degree is providing 7 days of 3D global ocean forecasts updated daily and ocean analysis updated weekly. The time series is aggregated in time in order to reach a two full year's time series sliding window. This product includes daily mean files of temperature, salinity, currents, sea level and ice parameters from the top to the bottom of the Ocean over the Global Ocean. It also includes 2-hourly mean surface fields for temperature, currents and sea level.
Spatial resolution	0.08 degree
Vertical coverage	from -5500.0 m to 0.0 m
Temporal resolution	Daily mean, hourly mean
Update frequency	Daily
Production unit	GLO-MERCATOR-TOULOUSE-FR

DATASET #4

Product name	**GLOBAL OCEAN BIOGEOCHEMISTRY NON-ASSIMILATIVE HINDCAST (PISCES)**
Product identifier	GLOBAL_REANALYSIS_BIO_001_018
Link	`http://marine.copernicus.eu/services-portfolio/access-to-products/?option=com_csw&view=details&product_id=GLOBAL_REANALYSIS_BIO_001_018` (last accessed on 15-12-2017)
Short description	Biogeochemistry non assimilative hindcast simulation GLOBAL_REANALYSIS_BIO_001_018 over period 1998 - 2014. Outputs are delivered as monthly mean files with Netcdf format (CF/COARDS 1.5 convention) on the native tripolar grid (ORCA025) at 1/4°resolution with 75 vertical levels. This simulation is based on the PISCES biogeochemical model. It is forced offline at a daily frequency by the equivalent of the GLOBAL-REANALYSIS-PHYS-001-009 physics product.
Spatial resolution	0.25 degree
Vertical coverage	from -5500.0 m to 0.0 m
Temporal resolution	Daily mean
Update frequency	Daily
Production unit	GLO-MERCATOR-TOULOUSE-FR

DATASET #5

Product name	**ATLANTIC - EUROPEAN NORTH WEST SHELF - OCEAN BIOGEOCHEMISTRY ANALYSIS AND FORECAST**
Product identifier	NORTHWESTSHELF_ANALYSIS_FORECAST_BIO_004_002_b

Link http://marine.copernicus.eu/services-portfolio/access-to-products/?option=com_csw&view=details&product_id=NORTHWESTSHELF_ANALYSIS_FORECAST_BIO_004_002_b (last accessed on 15-12-2017)

Short description The Forecasting Ocean Assimilation Model Atlantic Margin model (FOAM AMM7) is a coupled hydrodynamic-ecosystem model, nested in a series of one-way nests to the Met Office global ocean model. The hydrodynamics are supplied by the Nucleus for European Modelling of the Ocean (NEMO) with the 3DVar NEMOVAR system used for the assimilation of sea surface temperature data. This is coupled to the European Regional Seas Ecosystem Model (ERSEM), developed at Plymouth Marine Laboratory (PML). ERSEM based models have been used operationally to forecast biogeochemistry in the region for a number of years.

Spatial resolution 0.11 degree

Vertical coverage from -5000 m to 0 m

Temporal resolution Daily mean

Update frequency Daily

Production unit NWS-METOFFICE-EXETER-UK

DATASET #6

Product name **ATLANTIC- EUROPEAN NORTH WEST SHELF- OCEAN BIOGEOCHEMISTRY REANALYSIS FROM METOFFICE**

Product identifier NORTHWESTSHELF_REANALYSIS_BIO_004_011

Link http://marine.copernicus.eu/services-portfolio/access-toproducts/?option=com_csw&view=details&product_id=NORTHWESTSHELF_REANALYSIS_BIO_004_011 (last accessed on 15-12-2017)

Short description The reanalysis is based upon the Forecasting Ocean Assimilation Model 7km Atlantic Margin Model (FOAM AMM7).This is a hydrodynamic model of the North West European shelf forced at the surface by ERA-interim winds, atmospheric temperature, and precipitation fluxes. Horizontal boundary conditions were provided by the NOC global reanalysis prior to 1989 and by the GloSea reanalysis thereafter. Boundary conditions in the Baltic Sea came from the IOM-GETM model and CMEMS-BALTICSEA ANALYSIS_FORECAST_PHYS_003_006. E-Hype data were used for river inputs. Hydrodynamic calculations were performed by the Nucleus for European Modelling of the Ocean (NEMO) system, while the 3DVar NEMOVAR system was used for the assimilation of sea surface temperature data. Physical outputs are provided both as monthly means and as daily 25 hour, edited, averages.

Spatial resolution 0.11 degree

Vertical coverage from -5000.0 m to 0.0 m

Temporal resolution Daily mean

Update frequency Daily

Production unit NWS-METOFFICE-EXETER-UK

System 3 – Habitat monitoring

Variation in key environmental parameters was measured fortnightly (May 2015 - June 2016) at a traditional longline mussel farm "Glencoe Shellfish" in Loch Leven (56.7097 N, -5.0292 W; Scotland, UK). Temperature (°C) and salinity (psu), from vertical CastAway CTD profiles, and chl-a concentration (mg m^{-3}), using fluorometry, were measured at one, three, five, and nine meters depth along the rope (Michalek *manuscript in preparation*).

Supplementary Methods

Mytilus shell shape was analysed through an elliptic Fourier analysis (EFA) of outlines[15–17]. This geometric morphometrics approach[18,19] was performed in order to examine shell shape variation both within and between groups of individuals. As other modern morphometrics approaches, it considers outlines as a whole, taking into account all the geometrical relationships of the input data.

EFA is a powerful method to extract this geometric information and has been implemented on the concept of Fourier series: to decompose a periodic function into a sum of more simple trigonometric functions, such as sine and cosine[17,20]. These simple functions have frequencies that are integer multiples, therefore they are harmonics of one another.

This approach fits Fourier series separately on the x and y coordinates of an outline, projected on the Cartesian plane, as a function of the curvilinear abscissa[16,17,20]. EFA is then used to extract the geometrical information from outlines, described as periodic functions[16,21], through their decomposition into the harmonic sum of trigonometric functions, called harmonics. Low-frequency harmonics approximate coarse-scale trends in the original outlines, while high-frequency harmonics fit their fine-scale variations[17]. These can be normalized to remove homothetic, translational or rotational differences between shapes and smoothed in order to remove outline noise during the digitization process[22]. Harmonic coefficients are then extracted and used as shape variables capturing shape information. The geometrical information contained in the outlines is thus quantified and can be analysed with classical multivariate tools (i.e. multivariate analysis of variance, principal component analysis and linear discriminant analysis).

EFA of outlines allows shape reconstruction from the numerical signature and this improved method has great advantages compared to more traditional approaches[17,19,22]: complex shapes can be fitted, outlines smoothed, starting points and coefficients can be normalised to remove homothetic, translational and rotational differences between outlines[17,19,21–23]. Shape analyses were carried out using the Momocs ("Morphometrics using R")[17] package with the R v3.3.0 software[24].

EFA of outlines: acquisition, processing and analysis (Supplementary Figure S1)

- Digital images of orthogonal lateral and ventral shell views (left valves) were acquired with a high-resolution digital camera (Nikon D3300 camera, fitted with Sigma 105mm f/28 EX DG Macro lens);
- Photographs were processes with an image analysis software (©Adobe Photoshop), centred and consistently aligned;
- Photographs were converted into black masks on a white background (greyscale, 8-bit) and only the shapes of intact shells were retained;
- Outlines were isolated, converted into a list of $(x;y)$ pixel coordinates and used as input data.

Lateral and ventral views of each shell were processed independently and later combined for analysis:

- Prior to calculation of elliptic Fourier transforms, an outline alignment through geometric operations was directly performed on the list of coordinates. This *a priori* normalisation was required to avoid potential bias introduced by the numerical adjustment of shapes prone to bad alignment (usually circular or with bilateral symmetry). Indeed, for mussel outlines, "consuming" their first harmonic in order to normalise higher rank harmonics[20], determined a poor numerical alignment, resulting in not homologues elliptic Fourier descriptors[21,22];
- Outlines were first smoothed to remove any noise introduced during the digitization process, centred and outline coordinates were rescaled by their centroid size;
- Equal number of points were sampled along each outline (1000 pseudo-landmarks);
- Point configurations were aligned through a Procrustes superimposition[20,25] and starting points normalised;
- An EFA was then computed on the resulting coordinates from shapes invariant to outline size, rotation and position;
- After preliminary calibration, through inspection of i) the outline reconstruction efficiency, ii) the deviation from the optimal fit and iii) the spectrum of harmonic Fourier power, seven harmonics were chosen to encompass 98% of the total harmonic power[17,23];
- Four coefficients per harmonic (28 descriptors) were extracted for each outline and used as variables quantifying the geometrical information[20,21].

The shape information contained in the outlines was then quantified and analysed with classical multivariate tools:

- Principal component analysis (PCA), with a singular value decomposition method, was performed on the matrix of coefficients, without rescaling, in order to define axes capturing the most of shape variation among individuals[26,27];
- The first 10 PCs accounting for the 97% of outline variability were used as new shape variables;
- Multivariate Analysis of Variance (MANOVA) was performed on the new shape variables to test for a significant effect of location of origin and shell size on shape variances[20,26,27];
- Linear discriminant analysis (LDA) was performed on shape variables, with a leave-one-out cross-validation procedure, in order to evaluate whether the linear combination of shape features (discriminant function) was able to discriminate between *Mytilus* species. *A priori* classification probabilities were set to be proportional to group sizes and Wilks' λ was calculated to test for significant discriminations. Discriminant coefficients were estimated in order to identify shell shape features that optimized the between-species differences "relative" to the within-species variation[20,26];
- Shape differences at the extremes of the morphospace were visualised with representation of mean shapes, deformation grids[28] and iso-deformation lines, which were obtained though mathematical formalisation of thin plate splines (TPS) analysis[25];
- The first five shape variables (PCs), describing distinguishable shell features along the mussel outlines, were then analysed with generalized additive mixed models (GAMMs) in order to identify relationships between mussel shape variation and environmental gradients.

Supplementary References

1. Hilbish, T., Carson, E., Plante, J., Weaver, L. & Gilg, M. Distribution of *Mytilus edulis*, *M. galloprovincialis*, and their hybrids in open-coast populations of mussels in southwestern England. *Mar. Biol.* **140**, 137–142 (2002). DOI 10.1007/s002270100631.
2. Kijewski, T. *et al.* Distribution of *Mytilus* taxa in European coastal areas as inferred from molecular markers. *J. Sea Res.* **65**, 224–234 (2011). DOI 10.1016/j.seares.2010.10.004.
3. Kijewski, T., Wijsman, J. W., Hummel, H. & Wenne, R. Genetic composition of cultured and wild mussels *Mytilus* from The Netherlands and transfers from Ireland and Great Britain. *Aquac.* **287**, 292–296 (2009). DOI 10.1016/j.aquaculture.2008.10.048.
4. Yarra, T., Gharbi, K., Blaxter, M., Peck, L. S. & Clark, M. S. Characterization of the mantle transcriptome in bivalves: *Pecten maximus*, *Mytilus edulis* and *Crassostrea gigas*. *Mar. Genomics* **27**, 9–15 (2016). DOI 10.1016/j.margen.2016.04.003.
5. Tyler-Walters, H. *et al.* Descriptions of scottish Priority Marine Features (PMFs). Scottish Natural Heritage commissioned report No. 406. Tech. Rep. 406 (2016).
6. Stuckas, H. *et al.* Combining hydrodynamic modelling with genetics: can passive larval drift shape the genetic structure of Baltic *Mytilus* populations? *Mol. Ecol.* **26**, 2765–2782 (2017). DOI 10.1111/mec.14075.
7. Brooks, S. J. & Farmen, E. The distribution of the mussel *Mytilus* species along the Norwegian coast. *J. Shellfish. Res.* **32**, 265–270 (2013). DOI 10.2983/035.032.0203.
8. Breusing, C. *Population genetics and morphometric variation of blue mussels in the western Baltic Sea.* (Master Thesis. GEOMAR Helmholtz Centre for Ocean Research Kiel, 2012).
9. Väinölä, R. & Strelkov, P. *Mytilus trossulus* in Northern Europe. *Mar. Biol.* **158**, 817–833 (2011). DOI 10.1007/s00227-010-1609-z.
10. Stuckas, H., Stoof, K., Quesada, H. & Tiedemann, R. Evolutionary implications of discordant clines across the Baltic *Mytilus* hybrid zone (*Mytilus edulis* and *Mytilus trossulus*). *Hered.* **103**, 146–156 (2009). DOI 10.1038/hdy.2009.37.
11. Mathiesen, S. S. *et al.* Genetic diversity and connectivity within *Mytilus* spp. in the subarctic and Arctic. *Evol. Appl.* **10**, 39–55 (2017). DOI 10.1111/eva.12415.
12. Bierne, N. *et al.* Introgression patterns in the mosaic hybrid zone between *Mytilus edulis* and *M. galloprovincialis*. *Mol. Ecol.* **12**, 447–461 (2003). DOI 10.1046/j.1365-294X.2003.01730.x.
13. Kijewski, T. K., Zbawicka, M., Väinölä, R. & Wenne, R. Introgression and mitochondrial DNA heteroplasmy in the Baltic populations of mussels *Mytilus trossulus* and *M. edulis*. *Mar. Biol.* **149**, 1371–1385 (2006). DOI 10.1007/s00227-006-0316-2.
14. Dias, P. J. *et al.* Survey of mussel (*Mytilus*) species at Scottish shellfish farms. *Aquac. Res.* **40**, 1715–1722 (2009). DOI 10.1111/j.1365-2109.2009.02274.x.
15. Giardina, C. R. & Kuhl, F. P. Accuracy of curve approximation by harmonically related vectors with elliptical loci. *Comput. Graph. Image Process.* **6**, 277–285 (1977). DOI 10.1016/S0146-664X(77)80029-4.
16. Kuhl, F. P. & Giardina, C. R. Elliptic Fourier features of a closed contour. *Comput. Graph. Image Process.* **18**, 236–258 (1982). DOI 10.1016/0146-664X(82)90034-X.
17. Bonhomme, V., Picq, S., Gaucherel, C. & Claude, J. Momocs : outline analysis using R. *J. Stat. Softw.* **56**, 1–24 (2014). DOI 10.18637/jss.v056.i13.
18. Rohlf, F. & Marcus, L. F. A revolution in morphometrics. *Trends Ecol. & Evol.* **8**, 129–132 (1993). DOI 10.1016/0169-5347(93)90024-J.
19. Adams, D. C., Rohlf, F. J. & Slice, D. E. Geometric morphometrics: ten years of progress following the ”revolution”. *Italian J. Zool.* **71**, 5–16 (2004). DOI 10.1080/11250000409356545.
20. Claude, J. *Morphometrics with R* (Springer, 2008).
21. Rohlf, F. J. & Archie, J. W. A comparison of Fourier methods for the description of wing shape in mosquitoes (Diptera: Culicidae). *Syst. Zool.* **33**, 302 (1984). DOI 10.2307/2413076.
22. Haines, A. J. & Crampton, J. S. Improvements to the method of Fourier shape analysis as applied in morphometric studies. *Palaeontol.* **43**, 765–783 (2000). DOI 10.1111/1475-4983.00148.

23. Crampton, J. S. Elliptic Fourier shape analysis of fossil bivalves: some practical considerations. *Lethaia* **28**, 179–186 (1995). DOI 10.1111/j.1502-3931.1995.tb01611.x.

24. R Core Team. R: A language and environment for statistical computing. (2016). URL `https://www.r-project.org/`.

25. Bookstein, F. L. *Morphometric tools for landmark data: geometry and biology* (Cambridge University Press, Cambridge, 1991).

26. Claude, M. Log-shape ratios, Procrustes superimposition, elliptic Fourier analysis: three worked examples in R. *Hystrix* **24**, 94–102 (2013). DOI 10.4404/hystrix-24.1-6316.

27. Vendrami, D. L. J. *et al.* RAD sequencing resolves fine-scale population structure in a benthic invertebrate: implications for understanding phenotypic plasticity. *Royal Soc. Open Sci.* **4**, 160548 (2017). DOI 10.1098/rsos.160548.

28. Thompson, D. W. *On Growth and Form* (Cambridge University Press, Cambridge, 1917).
